# Uncorrected Refractive Errors Among Children Attending Pediatric Ophthalmology Clinic at Security Forces Hospital, Makkah, Saudi Arabia

**DOI:** 10.7759/cureus.36234

**Published:** 2023-03-16

**Authors:** Ghaidaa Khouj, Albandari Alharbi, Waleed Alghamdi, Yahya Alzahrani, Amna Fallata

**Affiliations:** 1 Department of Medicine and Surgery, Umm Al-Qura University, Makkah, SAU; 2 Department of Optometry, Qassim University, Qassim, SAU; 3 Department of Ophthalmology, Security Forces Hospital, Makkah, SAU

**Keywords:** saudi arabia, ophthalmology, pediatric, astigmatism, hyperopia, myopia, refractive errors

## Abstract

Background: Refractive errors are globally one of the most prevalent ocular disorders among pediatrics. This study aimed to determine the pattern of uncorrected refractive errors among children attending pediatric ophthalmology clinics at Security Forces Hospital, Makkah, Saudi Arabia.

Methods: This was a retrospective cohort clinic-based study including the records of children attending the pediatric ophthalmology clinic at Security Forces Hospital, Makkah, Saudi Arabia, between July 2021 and July 2022 who were diagnosed with refractive errors, ages between 4 and 14 years.

Results: A total of 114 patients were included in the study while 26 patients with other ocular disorders were excluded from the study. The mean age of children included in the study was 9.1 ± 2.9. The most prevalent refractive errors were hyperopic astigmatism (64%), followed by myopic astigmatism (28.1%), then myopia (5.3%), and hyperopia (2.6%). The overall uncorrected refractive error of this study was estimated to be 36%. No significant association was found between the factors of age and gender on the type of refractive errors (P-value > 0.05).

Conclusion: The most prevalent pattern of uncorrected refractive errors among children attending pediatric ophthalmology clinics at Security Forces Hospital, Makkah, Saudi Arabia was hyperopic astigmatism followed by myopic astigmatism. No differences were found between different age groups and between genders on the type of refractive errors. Implementation of adequate vision screening programs for school-aged children is essential to detect uncorrected refractive errors at an early age.

## Introduction

Refractive errors (REs) are the most common ocular disorders among children. It happens when the eye is unable to clearly focus on images from the outside world. It causes blurred vision, which can be severe enough to cause visual impairment [1]. REs can be divided into myopia (nearsightedness), hyperopia (farsightedness), and astigmatism [2]. According to the World Health Organization (WHO), approximately 153 million people over five years of age are visually impaired due to uncorrected or inadequately corrected REs, which are considered the second most common preventable cause of blindness among childhood disorders [3]. REs are associated with short and long-term complications among children and adults as they might affect their educational performance, capacity for learning, occupational opportunities, quality of life, and socioeconomic status for individuals, families, and communities [4]. Various studies have evaluated the prevalence of REs among children around the world, yet only a few studies have been conducted in Saudi Arabia [5]. In 2015, a retrospective cross-sectional study conducted in Medina, Saudi Arabia, included 1,893 children and concluded that the prevalence of uncorrected REs (UREs) was (34.9%), the prevalence of astigmatism (25.3%) which was higher than compared to that of anisometropia (7.4%), hyperopia (1.5%), and myopia (0.7%) [6]. Moreover, a cross-sectional study conducted among primary school students in Jazan, Saudi Arabia, found that the most prevalent REs was hyperopia (32.2%) followed by myopic astigmatism (31%) and then myopia (17%) [7]. Another study conducted among 1,319 preschool children at King Abdulaziz Medical City, Riyadh, Saudi Arabia, found that out of the 1,319 children, 60 children were diagnosed as having one or more types of REs, with an overall prevalence of 4.5% (4.2% in boys and 4.9% in girls) with no gender difference. The prevalence of different REs was as follows: myopia (2.5%), hyperopia (2.1%), astigmatism (2.5%), amblyopia (0.5%), and strabismus (0.5%) [8]. This reflects the importance of early screening and prompt management to avoid further visual impairment. Nevertheless, there is a lack of studies that investigate this major public health problem in Makkah, Saudi Arabia, which is one of the largest cities in the kingdom [9]. Therefore, estimating the pattern of uncorrected REs is important for both healthcare professionals and policymakers. Consequently, this retrospective study aimed to determine the pattern of uncorrected REs among children attending pediatric ophthalmology clinics at Security Forces Hospital, Makkah, Saudi Arabia.

## Materials and methods

Design and setting

This was a retrospective cohort clinic-based study to evaluate the pattern of uncorrected REs among children attending pediatric ophthalmology clinics in Security Forces Hospital, Makkah, Saudi Arabia, from July 2021 to July 2022.

Sampling procedure

**Figure 1:**
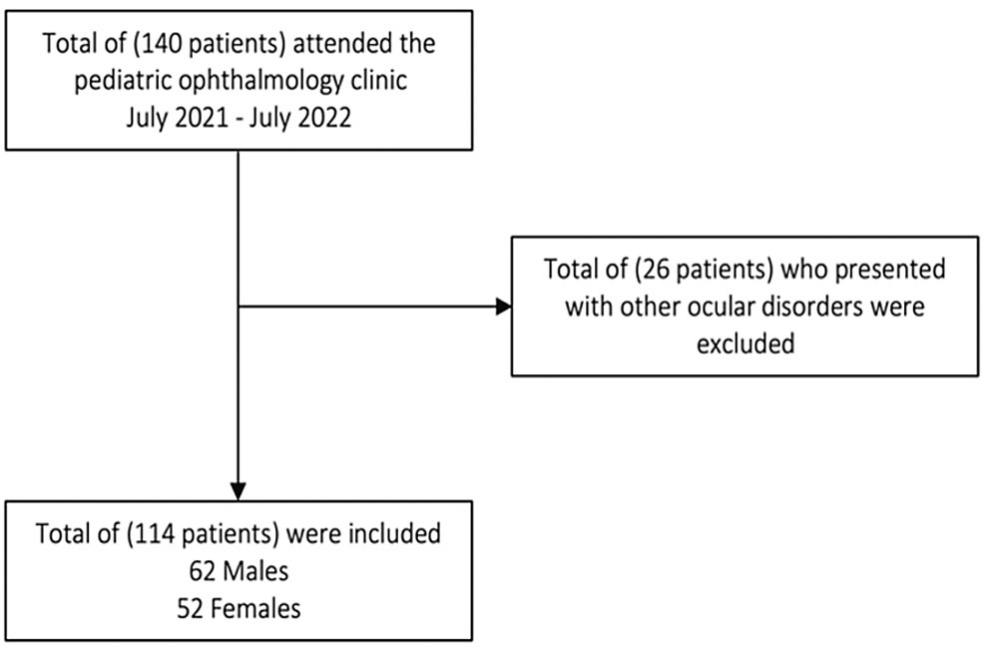
Diagram of the study population

The sample size for this study was calculated using a sample size formula, with the following parameters used to estimate sample size: population proportion = 50%, confidence level = 95%, and margin of error = 5%. We assessed a total of (140 patients) who were diagnosed with REs, ages between 4 and 14 years. A total of 26 patients who presented with other ocular disorders such as corneal opacity, congenital cataract, retinal disorders, optic nerve abnormality, RE associated with any mental delay or syndromes were excluded from the study (Figure 1).

Study tool

A complete ocular evaluation was performed, including visual acuity was measured using the Snellen chart, fundoscopic examination, subjective refraction, and cycloplegic refraction by administering two drops of 1% cyclopentolate to both eyes for at least 30 minutes prior to examination. Cyclopentolate drops were used to dilate the pupil of the eye and relax the ciliary muscles to assess refractive status by autorefractor and retinoscope. In the current study, the patients classified based on their REs as following: hyperopia, myopia, and astigmatism. Hyperopia was defined as the spherical equivalent of +2.00 diopters or greater in one or both eyes. Myopia also was defined as spherical equivalent of -0.75 diopters or greater in one or both eyes, and astigmatism as cylinder error equivalent of 0.50 diopters or greater and equivalent of 1.00 diopters or greater in one or both eyes. Relevant demographic data were documented from electronic files of the patients which included age, gender, type of REs, and history of wearing glasses, family history of REs, history of other ocular disorders. The data quality was carefully examined in the hospital by healthcare practitioners.

Ethical consideration

Official approval was obtained from the Institutional Review Board (IRB) of Security Forces Hospital, Makkah, Saudi Arabia, prior to data collection. IRB approval of research project number (0492-180722).

Statistical analysis

The data were collected, reviewed, and then fed to Statistical Package for Social Sciences version 21 (SPSS, IBM Company, NY, USA). All statistical methods used were two tailed with alpha level of 0.05 considered significant if P-value less than or equal to 0.05. The frequencies and percentage were used to express categorical variables including children’s demographic data, and family history of REs, while types of REs were graphed. The distribution and association of REs along with wearing glasses were assessed and analyzed using the cross tabulation. Further, the association of different types of REs with age groups and gender were investigated using chi-square test for significance and exact probability test if there were small frequency distributions.

## Results

**Figure 2:**
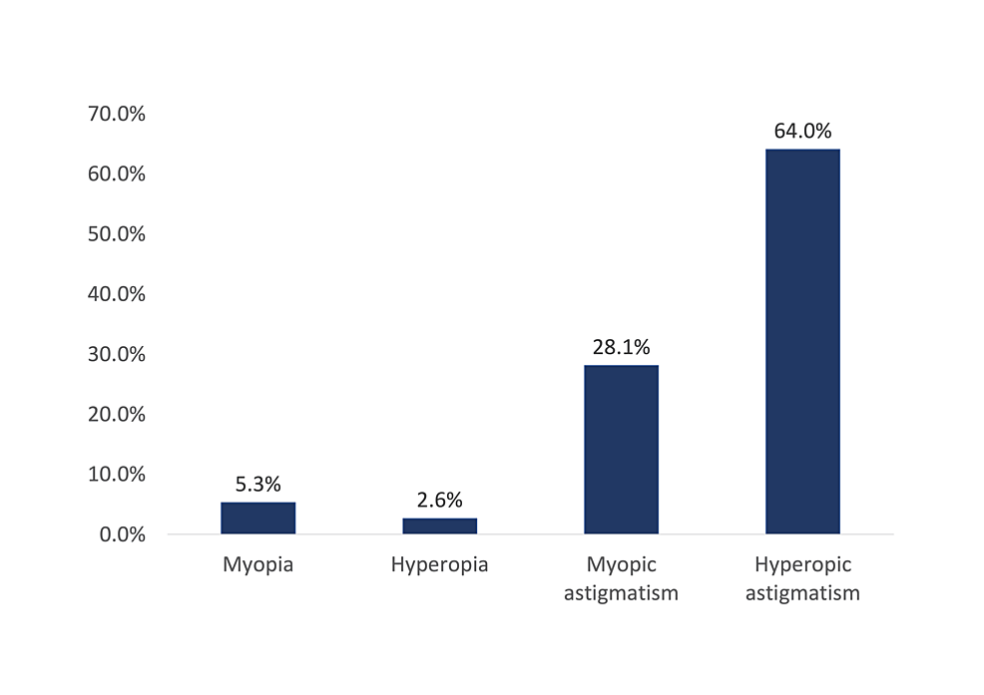
Types of refractive errors among children

A total of 114 children with REs and fulfilling the inclusion criteria were included. Children's ages ranged from 4 to 14 years with a mean age of 9.1 ± 2.9 years old. Sixty-two (54.4%) of the children were males and 10 (8.8%) reported having a family history of REs (Table 1). Figure 2 showed the distribution of types of uncorrected REs among study children with REs, Makkah, Saudi Arabia. In this study, 73 (64%) children had hyperopic astigmatism, 32 (28.1%) had myopic astigmatism, six (5.3%) had myopia, and three (2.6%) had hyperopia. Table 2 showed the number of children with UREs. Only 73 (64%) of the children presented with spectacles, with a high percentage was among children with hyperopic astigmatism (67.1%), followed by children with hyperopia (66.7%), myopic astigmatism (62.5%), and myopia (33.3%) with no significant association between wearing glasses and the type of refraction (P=0.424).

**Table 1:** Demographic data of children with refractive errors Standard deviation (SD), Percentage (%)

Demographic Data	Number	Percentage
Age in years		
4 - 7	33	28.9
8 - 10	48	42.1
11 - 14	33	28.9
Mean ± Standard deviation (SD)	9.1 ± 2.9	
Gender		
Male	62	54.4
Female	52	45.6
Family history of refractive errors		
No	36	31.6
Yes	10	8.8
Unknown	68	59.6

**Table 2:** Wearing glasses among children Probability test value (P-value), Percentage (%)

Refractive Errors	Wearing Glasses				P-value
	No		Yes		
	Number	Percentage	Number	Percentage	
Myopia	4	66.7%	2	33.3	0.424
Hyperopia	1	33.3%	2	66.7	
Myopic astigmatism	12	37.5%	20	62.5	
Hyperopic astigmatism	24	32.9%	49	67.1	
Total	41	36.0%	73	64.0	

The most diagnosed RE among young children (4-7 years) was hyperopic astigmatism (63.6%) which was also the most reported among other age groups (70.8% for 8-10 years and 54.5% for 11-14 years) with no statistical significance (P=0.260). Myopia was more among children aged 11-14 years (9.1%) (Table 3).

**Table 3:** Distribution of refractive errors by children age Probability test value (P-value), Percentage (%)

Refractive Errors	Age in Years						P-value
	4 - 7		8 - 10		11 - 14		
	Number	Percentage	Number	Percentage	Number	Percentage	
Myopia	0	0.0	3	6.3	3	9.1	0.260
Hyperopia	2	6.1	1	2.1	0	0.0	
Myopic astigmatism	10	30.3	10	20.8	12	36.4	
Hyperopic astigmatism	21	63.6	34	70.8	18	54.5	

Hyperopic astigmatism was the most diagnosed among male (64.5%) and female (63.5%) children. Myopia was diagnosed among (8.1%) of males versus (1.9%) of females while Myopic astigmatism was among (32.7%) of females versus (24.2%) of males (P=0.401) (Table 4).

**Table 4:** Distribution of refractive errors by children gender Probability test value (P-value), Percentage (%)

Refractive Errors	Gender				P-value
	Male		Female		
	Number	Percentage	Number	Percentage	
Myopia	5	8.1	1	1.9	0.401
Hyperopia	2	3.2	1	1.9	
Astigmatism	0	0.0	0	0.0	
Myopic astigmatism	15	24.2	17	32.7	
Hyperopic astigmatism	40	64.5	33	63.5	

## Discussion

UREs cause both immediate and long-term visual impairment in children and adults, which have an impact on academic performance, career choice, and socioeconomic status in later life [10]. Moreover, UREs are a significant issue among children in Saudi Arabia and worldwide, because of different factors including lack of personal and family awareness and recognition of the issue, inability to afford refractive services, and cultural barriers to compliance [11]. To the best of our knowledge, this is the first study to determine the pattern of uncorrected REs among children in Makkah city. The most common type of RE was hyperopic astigmatism (64%) of all, followed by myopic astigmatism (28.1%), myopia (5.3%), and hyperopia (2.6%). Conversely, a study conducted in Riyadh, Saudi Arabia found that the most prevalent RE among children is myopia (10.48%), followed by astigmatism (8.48%), hyperopia with astigmatism (4%), myopia with astigmatism (3.65%) and hyperopia (2.12%) [4]. Another study conducted in Jazan, Saudi Arabia demonstrated that hyperopia was the most common REs (32.2%) followed by myopic astigmatism (31%), myopia (17.2%), hyperopic astigmatism (16.1%), and mixed astigmatism (3.5%) [7]. Moreover, a study carried out in Al-Hassa, Saudi Arabia reported that myopia (65.6%) is the most prevalent REs among their population followed by myopic astigmatism (12.4%), hyperopic astigmatism (12%) and hyperopia (9.8%) [3]. On the other hand, previous studies in different cities in Saudi Arabia and countries worldwide have addressed the prevalence of REs among children as shown in (Table 5). Most importantly, myopia is the most common type of RE Worldwide as its prevalence varied between (4.9%-18.2%) [12]. The variances in results between these studies could be referred to the differentials in study design, method of conducting the refraction as well as the differences in population demography, genetics, and environmental factors [7]. In this study, regarding the age factor we found that children aged from (4-7 years) and (8-10 years) had hyperopic astigmatism as the most reported REs, while children aged from (11-14 years) had myopia with no statistical significance (P=0.260). Likewise, the gender factor in this study reported that hyperopic astigmatism was the most diagnosed RE among both males and females. Whereas myopia was slightly higher in males compared to females, and myopic astigmatism was more among females than males, but such differences were not statistically significant (P=0.401). Accordingly, the association between the composition of age and gender and the type of REs explores that no correlation was found. However, another study conducted in Medina, Saudi Arabia reported similar results to this study as the prevalence of REs showed no significant variation among groups. Excluding children with astigmatism as the result showed a significant association with age (P-value < 0.0001) [6]. Additionally, a study carried out in Qassim Province, Saudi Arabia found similar findings to this study regarding gender [5]. A previous study found an association between REs and family history of REs [7,13,14]. Hence, in this study family history of REs was assessed and no significant association was found. Consequently, additional investigations in this field to support the evidence of the presence or absence of this association are recommended.

This study has some limitations that must be acknowledged. First, the study was only conducted for a limited duration; a longer duration would provide larger data to be able to follow the patient in order to study the progress of REs, and its impact on educational achievement and equality of life. Furthermore, it was a clinic-based retrospective study; therefore, the generalizability of the results to all children of the city. Certainly, professional-based screening programs are recommended to address the issue of uncorrected REs in children to provide early detection and prompt treatment. Last, for future researchers, this is an important issue to investigate for other risk factors that may be associated with REs among children. In addition, we recommend more research on a larger sample of patients from different types of practice like government, military, and university hospitals.

**Table 5:** The prevalent type of refractive errors reported in different studies in Saudi Arabia and worldwide

	Country	Study design	Sample size	Year	The most prevalent type of refractive errors
Local [15]	Saudi Arabia - Taif [16]	Cross sectional school-based study	3,678	2022	Astigmatism
	Saudi Arabia - Uyoun Aljawa [17]	Cross sectional school-based study	417	2019	Hyperopia
	Saudi Arabia - Riyadh [18]	Cross sectional school-based study	414	2016	Myopia
	Saudi Arabia - Jazan [7]	Cross sectional school-based study	395	2015	Myopia
	Saudi Arabia - Medina [6]	Retrospective school-based study	1,893	2015	Astigmatism
	Saudi Arabia - Al-Hassa [3]	Cross sectional study	2,246	2011	Myopia
	Saudi Arabia - Qassim [5]	School-based study	5,176	2010	Myopia
Global	Mexico - Toluca [19]	Cross sectional school-based study	317	2016	Myopia
	Iran - Dezful [20]	Cross sectional school-based study	1,375	2014	Hyperopia
	USA - Los Angeles [21]	Retrospective school-based study	1,007	2013	Hyperopia
	China - Shanghai [22]	Cross sectional school-based study	8,267	2013	Myopia
	South Korea - Jeolla [23]	Cross sectional school-based study	1,079	2013	Myopia
	Turkey - Eskisehir [24]	Cross sectional school-based study	557	2011	Myopia
	Iran - Bojnourd [25]	Cross sectional school-based study	1,551	2010	Astigmatism

## Conclusions

In conclusion, this study estimates the most common type of uncorrected REs among children with REs attending pediatric ophthalmology clinics at Security Forces Hospital, Makkah, Saudi Arabia. The exact of 73 (64%) children had Hyperopic astigmatism followed by 32 (28.1%) had myopic astigmatism. Also, this paper examines the impact of age and gender on the type of REs, and no associations were found. Consequently, these findings highlight the importance of an adequate screening program for school-aged children.
